# Proper Medical Education

**Published:** 1884-09

**Authors:** 


### EDITORIAL.

### Proper Medical Education.

In the *Polyclinic* of August 15th is a paper on this subject by Henry Leffman, M.D., read before the Medical Society of Philadelphia, which contains much that applies to dental education as well. "The necessity for a change in the methods of medical education has been recognized for a number of years, and in obedience to a developing public sentiment the colleges are, one by one, improving their course of study. With many, however, this has been but a patchwork." * * * "It seems that to attain efficiency in medical education we must reform it, not "indifferently," but "altogether."

This is perhaps as true of dental as medical education. It is true, however, that in dental education, as now conducted by our colleges, there is far more of practical instruction than has been usually given in medical colleges, improvement is being made, however, in these by the increase of hospital and clinical advantages.

Of the defects mentioned in the paper, "A notorious one is, the entire absence of gradation in the study; the beginner and the advanced student are given the same instruction. The result in most cases is that medical studies are limited to about one year, for during the first year the student loiters, and during the second year he 'crams.'"

This is a difficulty doubtless which all teachers in both medical and dental schools have experienced; and it can only be remedied by proper grading, and the sooner the colleges come to some uniform standard in this respect the better.

A graded course, with examinations from time to time, and promotion dependent upon these, is the best and probably the only way of securing efficient work of students during the first college year. While a few might not need such stimulus, the great majority will be vastly benefited by it, not only in the first, but in the second year as well.

In a graded course a *final* examination should be made upon no branch till the time of graduation.

"It is a mistake to insist on the same course of study for each student without reference to his intention as to future practice."

This applies, of course, to the study of medicine as a whole. Still there is an idea in the minds of some in the dental profession, that practice would be improved by a separation of the operative and prosthetic branches. While this may be practicable in some instances, it cannot in the present condition of the profession be of general applicability. Still it might not be amiss to make some further experiment in this direction.

The paper further says:

"In our classical and general scientific colleges the courses are varied to the purpose of the student; the civil, mining, and mechanical engineers, for instance, give attention to branches partly in common and partly distinct. A similar specialization, could, it seems to me, very properly obtain in medical studies. * * * What advantage may accrue from high specialization is well seen by what has been accomplished in dentistry. This is undoubtedly a specialty of medicine; yet it has, owing to the precision with which students have been trained for it alone, grown into a distinct profession, with colleges, societies, State and national conventions, and examining boards, which latter, by the way, are in many cases more effective than those which have charge of regular medical practice. I cannot see any valid objections to giving ophthalmology, otology, and dermatology a similar distinct professional standing and independence; on the same principle upon which we have doctors of dentistry." This is a recognition of the status of dentistry that has not often been so plainly stated, or so frankly given, especially by physicians. It is, moreover, a testimony to the progress and advancement of the profession that is gratifying.

"The rapid extension and popularity of post-graduate courses are an indication of the deficiency of college instruction, and of the vigor of the specializing movement in medicine. It is from the organization of these schools that our colleges should take suggestions for the distribution of branches."

Many of those who graduate from our dental colleges sorely feel the need of further study and work, and instruction for that matter, to properly fit them for their future work; if not in respect to knowledge and skill, yet to give that confidence and self-reliance that is an important factor in all our doings of life.

"The question of preliminary examination is one that must be met fairly and courageously. It will be necessary to have it conducted in such a manner that it shall be something more than nominal. It is insinuated that it amounts to very little in some institutions, but we find little fault with these when we recall that some colleges in good standing do not make even the pretense of determining the fitness of the candidates."

This subject of preliminary examination for entering upon a course of professional study is on all hands eliciting attention and consideration. All admit the desirability and importance of a good preliminary education. But the difficulty of making such an examination thorough and just is brought as an objection. Why is such an objection valid here more than anywhere else? There is is not a literary or scientific college in the country, nor anywhere, that does not admit its pupils upon the ground of certain attainments having been made ,and this is determined usually by an examinaation, by just such a method as professional schools ought to adopt. But, says the objector, we are liable to be imposed upon. That, perhaps, is true, but it has no more force here than in thousands of other matters, where no one thinks of applying it. Impositions abound everywhere, and it is the part of wisdom to detect, and of integrity to put them aside when detected.

One of the advance steps made by the Association of Dental College Faculties is recommending a preliminary examination, to which doubtless all the profession will say AMEN.

